# Increased diversity of a cervical microbiome associates with cervical cancer

**DOI:** 10.3389/fonc.2022.1005537

**Published:** 2022-09-28

**Authors:** Natalia Zeber-Lubecka, Maria Kulecka, Bogusław Lindner, Ryszard Krynicki, Agnieszka Paziewska, Andrzej Nowakowski, Mariusz Bidzinski, Jerzy Ostrowski

**Affiliations:** ^1^ Department of Gastroenterology, Hepatology and Clinical Oncology, Centre of Postgraduate Medical Education, Warsaw, Poland; ^2^ Department of Genetics, Maria Sklodowska-Curie National Research Institute of Oncology, Warsaw, Poland; ^3^ Department of Gynaecological Oncology, Maria Sklodowska-Curie National Research Institute of Oncology, Warsaw, Poland; ^4^ Department of Cancer Prevention, Maria Sklodowska-Curie National Research Institute of Oncology, Warsaw, Poland

**Keywords:** cervical microbiome, chemoradiation therapy, 16S rRNA gene sequencing, lactobacillus, postmenopause

## Abstract

The cervical microbiome (CM) is a complex ecosystem that can change in response to gynecological cancers. We aimed to evaluate changes in the CM of patients who underwent chemoradiation (CRT) therapy for locally advanced cervical cancer. Before and after CRT, cervical swab samples were collected from 16 patients with squamous cell carcinoma of the cervix, and 30 healthy women. All samples were subjected to 16s rRNA-Seq analysis. In healthy premenopausal women the CM comprised mostly *Lactobacillus* (>90%); the CM community in samples from both pre- and postmenopausal pre-treatment cancer patients was heterogeneous, with a low proportion of *Lactobacillus* in younger cases. On the genus level, 27 and 11 taxa differentiated healthy controls from cancer patients in pre- and postmenopausal age groups, while 31 and 2 genera differentiated pre- and post-radiation samples and pre-radiation and the follow-up samples, respectively. Microbiome diversity was significantly higher in pre-treatment patients than in healthy controls. The results reveal significant alterations in the CM of cervical cancer patients relative to that in healthy controls; these changes were more striking after CRT. However, further research is needed to determine whether alteration of the CM offers new therapeutic options.

## Introduction

Collectively, human microbiotas and their genomes are called “the human microbiome”. There is high variation in the microbiome communities at specific body sites and within individuals (1, 2). Microbial communities in the gastrointestinal tract train the immune system, protect against opportunistic pathogens, and harvest nutrients and energy from non-digestible carbohydrates (3). Like the gut microbiome, the cervicovaginal microbiome is a complex ecosystem that broadly acts to maintain a woman’s genital tract health and comprises 20–140 bacterial species, of which *Lactobacillus* species are the most abundant. The lactobacilli abundance is age-related and depends on the estrogen levels, contributing to production of organic acids, primarily lactate, from glucagon deposited in the mature vaginal epithelium. In turn, acidifying of the vaginal ecosystem provides a protective barrier against viral and bacterial pathogens (4–6). After menopause, the lack of estrogen lead to a decrease in *Lactobacillus* and an increase in anaerobic bacteria in the vaginal flora (7). However, various other factors, including ethnicity, sexual activity, hygiene habits, lactation, and dietary factors can also affect the composition of the vaginal microbiota (1, 8–12). Alterations caused by immune regulation and inflammation lead to, or are associated with, several gynecological pathologies, including bacterial vaginosis (BV). BV represents a shift from a *Lactobacillus*-dominant to a polymicrobial microbiome with an increased abundance of anaerobic bacteria, such as *Gardnerella vaginalis, Peptostreptococcus anaerobius*, and *Porphyromonas uenonis*, and is associated with the progression of *Human papillomavirus* (HPV)-related cervical intraepithelial neoplasia and cancer risk (4, 13–20).

Curative or palliative radiation therapy (RT) and chemoradiation therapy (CRT) for gynecologic cancers may alter the composition of the cervical microbiome (CM) (21). A previous pilot study showed a strong reduction in cervical bacterial load after RT, with no changes in alpha or beta diversity (4). Another study reported that on the genus level, 13 phylogroups differentiated pre-RT from post-RT samples, and showed that most post-RT microbiota communities did not overlap with the communities in the normal microbiome. Yet another study reported a trend toward lower microbial richness in healthy samples relative to samples from patients with gynecological cancer (22).

Here, we used 16S rRNA gene amplicon sequencing to evaluate changes in the CM of patients who underwent CRT for locally advanced cervical cancer. While the lactobacilli abundance dominated CM of premenopausal healthy women, both pre- and postmenopausal cancer patients showed higher CM diversity than those found in both groups of healthy controls. The cancer-related composition of CM was further altered by CRT.

## Materials and methods

### Patients and controls

Sixteen patients (median age, 56 years; range, 25-62 years) with squamous cell carcinoma of the cervix who were indicated for primary RT were recruited. Of them, 6 patients (age 25–54) were classified as premenopausal or perimenopausal and 10 others (age 54-62) as postmenopausal, according to STRAW guidelines (23). Before treatment, the clinical stage was determined according to the International Federation of Gynecology and Obstetrics (FIGO) staging system (24). All patients received definitive RT comprising external beam radiation therapy (EBRT) and brachytherapy (ICBT). EBRT was performed with a total dose of 46 or 50 Gy, administered to the whole pelvis, followed by three fractions of ICBT (each as a single dose of 7.5 Gy) delivered to the cervix and residual tumor in all but one patient. All but two patients received concomitant cisplatin-based chemotherapy (40 mg/m^2^ body surface area) once a week for 5–6 weeks. The patients were followed between 2.5 and 4 years, and two patients relapsed for more than 2.5 years after the end of treatment. Patient characteristics are shown in **Table 1**.

**Table 1:** Clinical characteristics of the enrolled patients with cervical cancer.

Age (years)	FIGO Staging	Smoking	Diabetes	HT	EBRT	ICBT	Cisplatin	Relapse
49	IIb	+	–	+	46 Gy	3x7,5 Gy	+	–
54	IIIb	+	–	–	50 Gy	2x7,5 Gy	+	–
55	IIb	–	+	+	46 Gy	3x7,5 Gy	+	–
54	IIb	–	–	+	46 Gy	3x7,5Gy	+	–
35	IIIb	–	–	+	46 Gy	3x7,5 Gy	–	–
62	IIa	+	+	+	46 Gy	3x7,5 Gy	+	–
62	IIb	+	–	–	46 Gy	3x7,5 Gy	+	–
40	IIIb	+	+	–	50 Gy	3x7,5 Gy	–	+
48	IIb	–	–	–	46 Gy	3x7,5 Gy	+	–
61	IIb	–	–	+	46 Gy	3x7,5 Gy	+	–
57	IIIb	–	–	–	50 Gy	3x7,5 Gy	+	–
60	IIb	+	–	+	46 Gy	3x7,5 Gy	+	–
58	IIIb	+	+	–	46 Gy	3x7,5 Gy	+	–
25	IIb	–	–	–	46 Gy	3x7,5 Gy	+	–
62	IIb	–	–	+	46 Gy	3x7,5 Gy	+	–
61	IIIb	–	–	–	50 Gy	3x7,5 Gy	+	+

FIGO, International Federation of Gynecology and Obstetrics; HT, Hypertension; EBRT, radiation therapy; ICBT, brachytherapy.+, positive; -, negative.

Cervical swab samples were collected aseptically from each patient using 4N6FLOQSwabs™ (Thermo Fisher Scientific, USA) 1 day before starting EBRT, and then again on the day on which the last fraction of brachytherapy was given. In addition, swab samples were taken from nine and seven patients 3 and 6 months later, respectively. Exclusion criteria included other gynecologic cancers (ovarian, endometrial, or vulvar cancer), pregnancy, and treatment with antibiotics or antifungals for at least 2 months before enrollment.

The control group comprised 30 healthy women (median age, 51 years; range, 27–59 years) who were recruited under the human papillomavirus-based cervical cancer screening program. They were HPV negative and had normal Pap smears; none of them was on hormone replacement therapy.

All enrolled cervical cancer patients and controls were Polish Caucasians. Each swab sample was transferred to a clean collection tube and stored at −80°C within 1 hour of collection.

### DNA extraction and 16S rRNA sequencing

Microbial genomic DNA was extracted from swab samples using a PureLink™ Microbiome DNA Purification Kit, according to the manufacturer’s instructions. The concentration of bacterial DNA was measured using a Nanodrop ND-1000 spectrophotometer (Thermo Fisher Scientific, USA). DNA was stored at -20°C until further analysis. Bacterial 16S rRNA libraries were prepared using an Ion 16S™ Metagenomics Kit (which allows a consensus view across six regions: V2, V3, V4, V6-7, V8, and V9) and an Ion Plus Fragment Library Kit, as previously described (25–28). Next, 16S rRNA gene libraries were sequenced on an Ion Torrent Personal Genome Machine (PGM) platform (Thermo Fisher Scientific, USA) using Ion PGM™ Hi-Q™ View OT2 and Ion PGM™ Hi-Q™ View Sequencing Kits.

### Statistical and bioinformatic analysis

Unmapped BAM files were converted to FASTQ using Picard’s SamToFastq (29). Additional steps of the analysis were performed using Mothur version 1.38 software (30). FASTQ files were converted to the FASTA format. For analyses, only the sequences that were 200–300 bases in length, with an average base quality of 20 in a sliding window of 50 bases, and a maximum homopolymer length of 10 were kept. Chimeric sequences were identified with the UCHIME algorithm using default parameters (31), with internal sequence collection as the reference database. Chimeric sequences were removed, and the remaining 16S rRNA sequences were classified using the Wang method and the SILVA bacterial 16S rRNA database (32) for reference (release 138), at an 80% bootstrap cut-off. *Lactobacillus* species identification was based on reclassification to Greengenes 13.8.9 database (33). The non-parametric Shannon diversity index and the Chao richness index were determined with Mothur. For further analysis, sequences classified as chloroplasts, mitochondria, unknown, Archaea, and Eukarya we removed as recommended (34). Counts on genus level were obtained with MEGAN5. Bray-Curtis indices and PCoA analysis were performed with MEGAN5 (35). Differences in the abundance of taxa between groups were assessed with ANCOMB-BC (36). Mann-Whitney U-test was applied to determine differences in diversity indices between control samples and pretreatment samples and Wilcoxon paired test was used to determine statistically significant differences between paired patient samples. FDR-adjusted p-values smaller than 0.05 were considered significant. Bacteria respiratory status (Aerobic/Anaerobic/Facultatively Anaerobic) was obtained with BugBase, using Greengenes taxonomic annotations (37).

## Results

A total of 70 cervical swab samples were collected; of these, 40 samples were taken from 16 cancer patients: one sample from each patient, a day before starting EBRT and one after the last fraction of brachytherapy was given, and additional samples were taken from 9 and 7 patients 3 and 6 months later (the follow-up samples), respectively. From each of the 30 healthy controls, one sample was obtained. While all healthy women were HPV negative, the HPV status of cancer patients was not analyzed. Bacterial DNA extracted and purified from the swab samples was used for PCR amplification of bacterial 16S rRNA gene hyper-variable regions. Prepared libraries were sequenced using the PGM platform. For all but one sample with only 5083 reads, sequencing generated 37104-676457 (median, 127741; mean, 178047) reads; all of them passed quality control and 94–100% (median, 100%) of sequences were classified using SILVA database version 138 as a reference, and then assigned to *Bacteria* and *Archaea* taxa. The number of sequencing reads differentiated control samples (range, 77074-676457; median, 223136) from pre-treatment cancer samples (range, 37104-288927; median, 100536), p-value in Wilcoxon’s test=2.082e-05. Of 814 taxa (115 found in more than 0.01% of reads), 471 (72) were detected in both groups, while 145 (22) and 198 (38) were detected only in healthy controls and cervical cancer patients, respectively.

**Figure 1:**
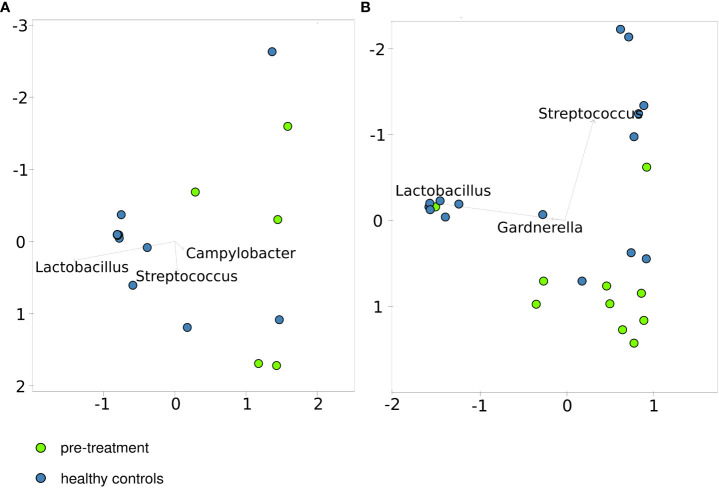
Principal coordinates analysis based on the Bray–Curtis index for healthy controls and patients before treatment. - pre-menopausal **(A, B)** – postmenopausal cases and controls.

Of 46 women included in this study, 21 (6 cancer patients and 15 healthy controls) were premenopausal women and 25 (10 cancer patients and 15 healthy controls) postmenopausal women. After excluding one patient with the low number of reads in the pre-treatment sample and one post-treatment sample with <75% of classified sequences, similarities between the CM community structures of the healthy control and pre-treatment cervical cancer groups, representing either premenopausal or postmenopausal cases and controls, were evaluated using principal coordinate analysis (PCoA) of Bray–Curtis distances. Twelve of the 15 CM samples from premenopausal healthy women and 7 CM samples from postmenopausal healthy women clustered together (**Figures 1A, B**), and both pre- and postmenopausal healthy groups were clearly separated from the corresponding cancer groups.

**Figure 2:**
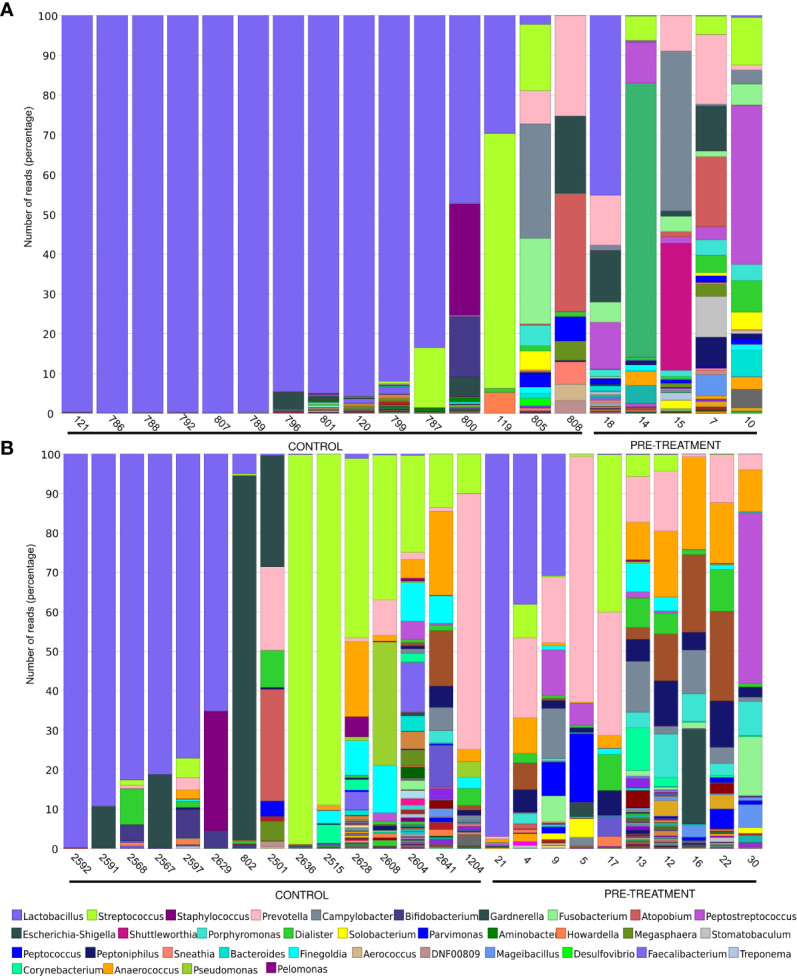
Microbiome composition on the genus level in women pre- **(A)** and postmenopause **(B)**. Unclassified reads on this level are not included. The legend includes only the most abundant bacteria.

The lactobacilli abundance depends on estrogen levels [34]. The proportion of *Lactobacillus* in the CM ranged from 0.01% to 99.91% (median, 94.99%) and from 0.04% to 45.27% (median, 0.12%) in premenopausal controls and cases (p-value 0.005), while in postmenopausal groups *Lactobacillus* abundance ranged from 0.022% to 99.67% (median, 1.08%) and from 0.005% to 96.87% (median, 0.07%) in controls and cases, respectively (p-value 0.1) (**Figures 2A, B**).

Eight (62%), 1 (8%), and 4 (30%) of 13 premenopausal healthy women with noticeable lactobacilli levels were dominated by *L. crispatus* (range, 46-94%), *L. iners* (100%) and *L. unclassified* (6-100%), respectively, and 7 postmenopausal healthy women were dominated by *L. iners* (2 women), *L. unclassified* (3 women) and *L. crispatus* (2 women). One woman had also noticeable levels (46%) of *L. delbrueckii*. All 4 CM of cancer patients with noticeable lactobacilli levels were dominated by *L. iners* (>99.9% of all lactobacilli reads).

**Figure 3:**
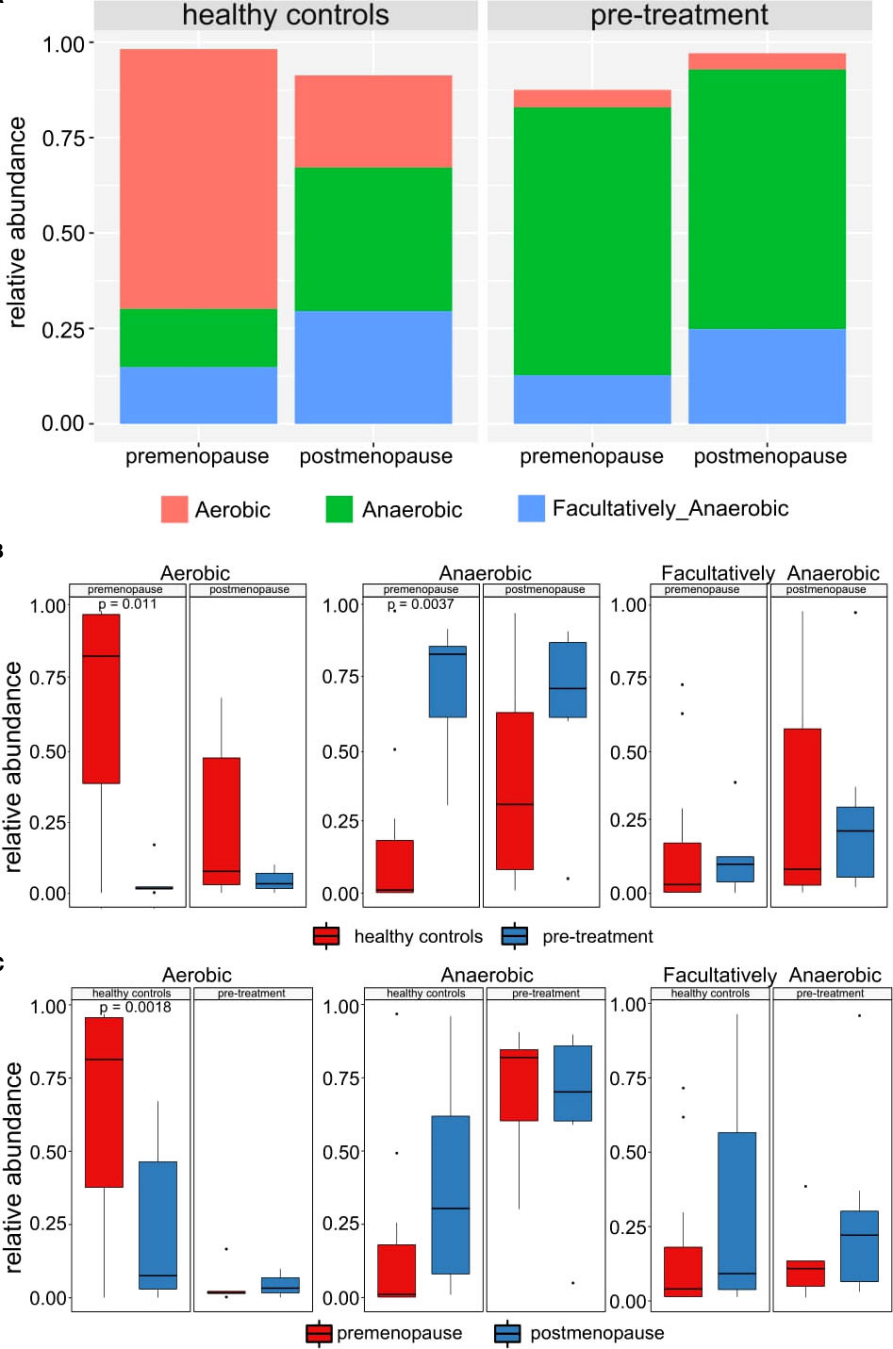
**(A)** Mean relative abundance of anaerobic, aerobic and facultatively anaerobic bacteria in healthy controls and pre-treatment patients. **(B)** Differences between abundances of anaerobic, aerobic and facultatively anaerobic bacteria in healthy controls and pre-treatment patients. **(C)** Differences between abundances of anaerobic, aerobic and facultatively anaerobic bacteria in pre- and post-menopausal women.

We next analyzed proportion of aerobic and anaerobic bacteria among the studied groups of patients and controls. While significantly lower (p-value=0.0027) and higher (p-value=0.013) percentage of anaerobic and aerobic bacteria, respectively, was found in premenopausal compared to postmenopausal healthy women, no similar differences were observed between pre- and postmenopausal cancer patients (**Figures 3A, C**). In addition, significantly lower (p-value=0.011) and higher (p-value=0.0037) percentage of aerobic and anaerobic bacteria, respectively, was found in premenopausal cancer patients compared to premenopausal control. In comparison of postmenopasal cancer patients and controls similar trends were observed but the differences did not reach statistical significance (**Figures 3A, B**).

**Figure 4:**
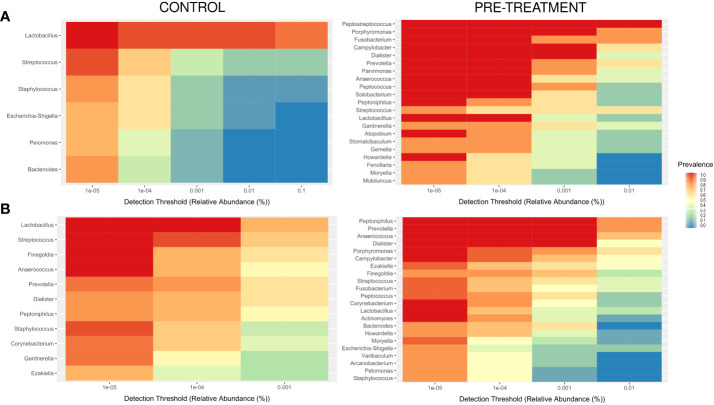
Core microbiome, with a minimum prevalence of 70% and a detection level of 0.00001% in pre- **(A)** and postmenopause **(B)** patients and controls.

A “core” CM of premenopausal healthy women (genera with a prevalence >70% and a detection level >0.001%) comprised six genera, one of which were present in all samples, and a core CM of postmenopausal controls comprised 11 genera, 4 of which were present in all samples (**Figure 4B**). CM community structures in pre-treatment samples from cancer patients were much more heterogeneous with the core CM which comprised 21 and 22 taxa, 14 and 9 of which were present in all samples in pre- and postmenopausal cases (**Figure 4**).

Next, we compared differences in taxonomic abundance between control individuals and pre-treatment cancer patients. To identify the specific bacterial taxa associated with cervical cancer, we compared the composition of the CM on the genus level, separately for pre- and postmenopausal women. Twenty seven and 11 genera differentiated healthy controls from cancer patients in pre- and postmenopausal age groups, respectively. Seven of these genera: *Porphyromonas, Fusobacterium, Peptococcus, Peptoniphilus, Moryella, Stomatobaculum*, and *Gemella* differentiated both groups and the first four are also highly abundant, with more than 10000 reads each (**Table 2**). Additional 12 and 1 differential genera are highly abundant in pre- and postmenopausal patients, respectively, including core genera for CM microbiota, *Lactobacillus* (in premenopausal patients) and *Gardnerella* (in postmenopausal samples). The number of differential taxa dropped to 18 and 9 in post-radiation samples in pre- and postmenopausal women respectively (**Supplementary Table S2**).

**Table 2:** Bacteria on genus level differentiating healthy controls and pre-treatment samples.

Genus	coef	se	test statistic	p-value	adj p-value	Age group	Abundant	AE/AN
*Arcanobacterium*	2.03	0.54	3.77	1.65E-04	6.90E-03	postmenopause		AN
*Fusobacterium*	3.65	1.01	3.63	2.84E-04	9.49E-03	postmenopause	yes	AN
*Gardnerella*	-4.12	1.20	-3.42	6.19E-04	1.72E-02	postmenopause	yes	AN
*Lentilitoribacter*	-1.17	0.39	-2.97	2.94E-03	4.46E-02	postmenopause		AE
*Gemella*	-2.01	0.67	-3.00	2.68E-03	4.46E-02	postmenopause		AN (F)
*Moryella*	2.63	0.85	3.09	2.02E-03	3.98E-02	postmenopause		AN
*Mesorhizobium*	-1.71	0.44	-3.94	8.29E-05	4.62E-03	postmenopause		AE
*Stomatobaculum*	2.45	0.80	3.07	2.15E-03	3.98E-02	postmenopause		AN
*Peptococcus*	2.74	0.89	3.08	2.10E-03	3.98E-02	postmenopause	yes	AN
*Peptoniphilus*	2.66	0.61	4.38	1.21E-05	1.01E-03	postmenopause	yes	AN
*Porphyromonas*	4.66	0.82	5.66	1.54E-08	2.58E-06	postmenopause	yes	AN
*Parvimonas*	5.23	1.04	5.05	4.46E-07	8.78E-06	premenopause	yes	AN
*Aminobacter*	-2.09	0.65	-3.20	1.35E-03	7.99E-03	premenopause		AE
*Atopobium*	3.99	1.45	2.74	6.09E-03	2.76E-02	premenopause	yes	AN
*Bulleidia*	3.17	1.02	3.09	1.98E-03	1.11E-02	premenopause		AN
*Acinetobacter*	-1.42	0.50	-2.85	4.32E-03	2.08E-02	premenopause		AE
*Peptoniphilus*	3.85	1.13	3.41	6.43E-04	4.46E-03	premenopause	yes	AN
*Anaerococcus*	4.11	0.91	4.53	5.97E-06	7.83E-05	premenopause	yes	AN
*Prevotella*	4.64	1.28	3.62	2.93E-04	2.47E-03	premenopause	yes	AN
*Gemella*	3.98	1.09	3.65	2.63E-04	2.38E-03	premenopause		AN (F)
*Campylobacter*	5.75	1.10	5.25	1.54E-07	4.54E-06	premenopause	yes	AE (M)
*Cutibacterium*	-1.22	0.47	-2.59	9.56E-03	4.18E-02	premenopause		AN (AEt)
*Moryella*	3.26	0.84	3.89	1.01E-04	1.08E-03	premenopause		AN
*Peptococcus*	4.15	0.88	4.72	2.37E-06	3.50E-05	premenopause	yes	AN
*Dialister*	4.09	0.84	4.86	1.17E-06	1.96E-05	premenopause	yes	AN
*Peptostreptococcus*	8.12	0.63	12.87	6.80E-38	8.03E-36	premenopause	yes	AN
*Porphyromonas*	5.81	0.74	7.89	3.09E-15	1.83E-13	premenopause	yes	AN
*Escherichia-Shigella*	-2.85	0.64	-4.43	9.28E-06	1.10E-04	premenopause	yes	AN (F)
*Pseudomonas*	-1.57	0.53	-2.99	2.83E-03	1.45E-02	premenopause	yes	AE
*Staphylococcus*	-2.63	0.81	-3.23	1.22E-03	7.56E-03	premenopause	yes	AN (F)
*Stomatobaculum*	4.77	1.27	3.75	1.76E-04	1.74E-03	premenopause		AN
*Fastidiosipila*	3.05	1.07	2.85	4.40E-03	2.08E-02	premenopause		AN
*Fenollaria*	3.76	1.15	3.27	1.08E-03	7.07E-03	premenopause		AN
*Rheinheimera*	-1.10	0.36	-3.01	2.59E-03	1.39E-02	premenopause		AE
*Fusobacterium*	5.69	1.07	5.32	1.05E-07	4.11E-06	premenopause	yes	AN
*Lactobacillus*	-4.98	1.46	-3.42	6.27E-04	4.46E-03	premenopause	yes	AN (AEt)
*Solobacterium*	4.76	0.92	5.16	2.51E-07	5.92E-06	premenopause	yes	AN
*Mobiluncus*	2.94	0.86	3.43	5.97E-04	4.46E-03	premenopause		AN

Coef - beta coefficient in ANCOM-BC log-linear model, se - standard error, test-statistic – test statistic for beta coefficient, p-value - p-value for beta coefficient, adj p-value - p-value adjusted for multiple comparison, AN, anaerobic; AE, aerobic; AN (AEt), aerotolerant anaerobic; AN (F), facultatively anaerobic; AE (M), microaerophilic.

**Figure 5:**
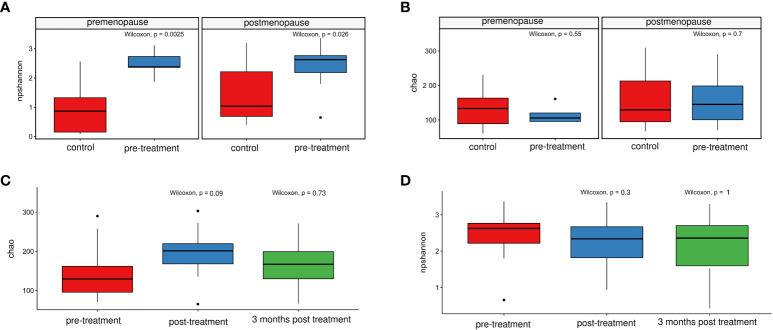
Comparison of the non-parametric Shannon indices for healthy controls and patients before treatment. **(A)** and for patients before and after treatment, and at 3/6 months post-treatment **(C)** Chao index- comparison in healthy controls and patients before treatment **(B)** and in patients before and after treatment, and at 3/6 months post-treatment **(D)**.

The observed microbiome diversity, as measured by the Shannon index (non-parametric), was significantly higher in pre-treatment patients than in healthy controls (**Figures 5A, B**) regardless of the cases and controls hormonal status. No significant differences were observed for species richness, as measured by the Chao index (**Figure 5B**), in both pre- and postmenopausal groups.

### Comparison of pre-radiation and post-radiation samples

**Figure 6:**
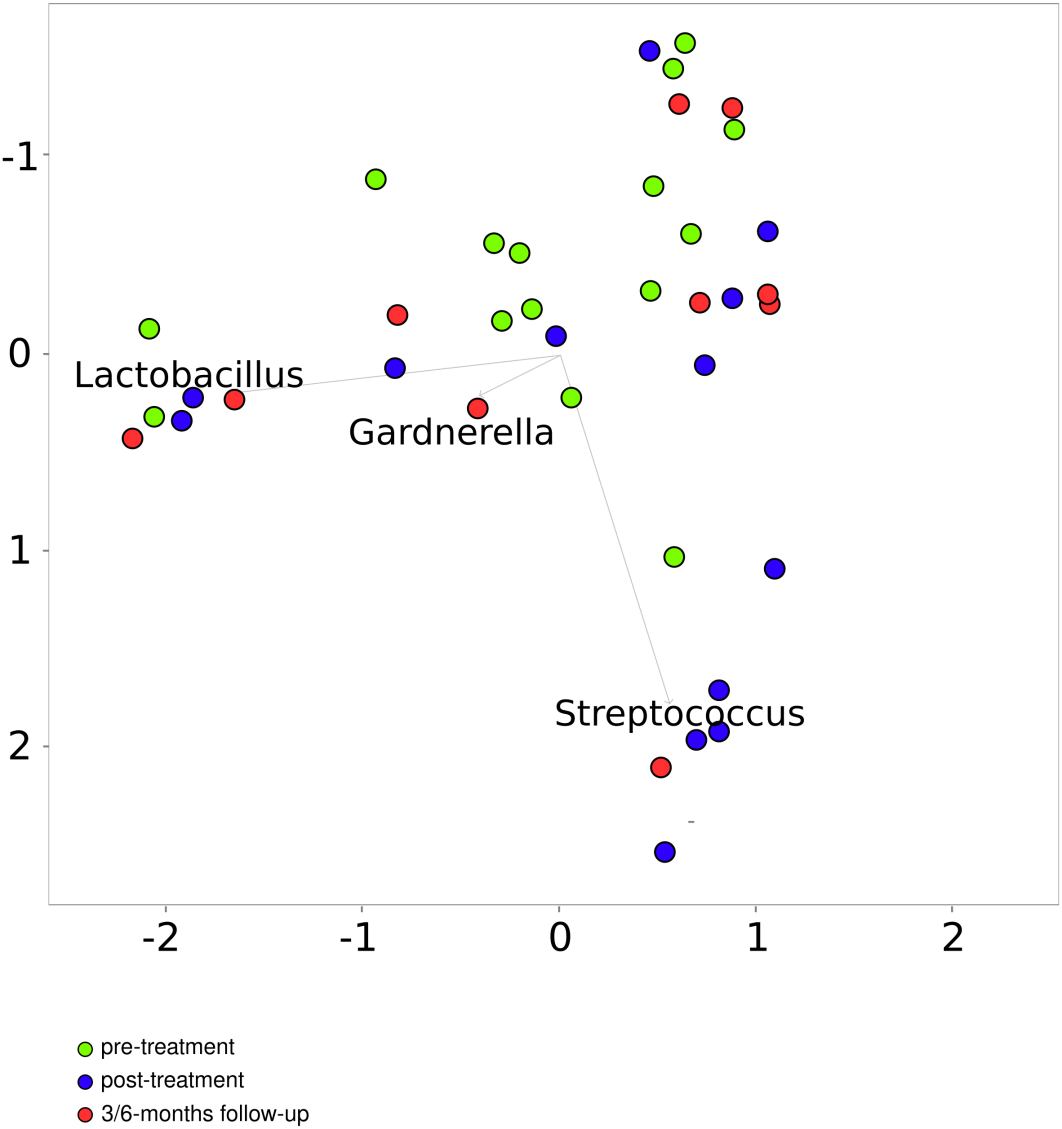
Principal coordinates analysis based on the Bray–Curtis index for patients before and after treatment.

The number of sequencing reads from pre-radiation samples (range, 37104-288927; median, 100536), samples collected immediately after the treatment (range 46747- 488737; median, 74075) and 3 and/or 6 months later (range 55295- 437511; median, 72950) did not differentiate pre-radiation and post-radiation samples. Of 669 taxa (148 found in more than 0.01% of reads) found in 40 samples of cancer patients, 376 (39) were detected in both pre- and post-radiation samples, while 69 (20), 155 (32), and 36 (0) were detected only in pre-treatment samples, samples collected immediately after the end ICBT and the follow-up samples, respectively. PCoA performed on the taxa data from the cancer patient samples, basing on Bray-Curtis distances, roughly differentiated pre-radiation from post-radiation samples, while differences were not noticeable between the post-treatment samples and follow-up samples (**Figure 6**).

On the genus level, 31 genera differentiated pre- and post-radiation samples, while only 2 differentiated pre-radiation and follow-up samples. Among the most abundant differentiating genera were *Streptococcus*, *Prevotella*, *Fusobacterium*, *Porphyromonas*, and *Finegoldia* (**Table 3**). There were no significant changes in CM diversity nor richness between pre-radiation samples and post-radiation samples, or the follow-up samples (**Figures 5C, D**).

**Table 3:** Bacteria on genus level differentiating pretreatment samples and posttreatment samples (after treatment and 3-month post-treatment).

Genus	coef II	se II	test statistic II	p-value II	adj p-value II	coef III	se III	test statistic III	p-value III	adj p-value III	Abundant	AE/AN
*Staphylococcus*	2.91	0.42	6.99	2.81E-12	5.14E-10	0.72	0.35	2.02	4.36E-02	3.07E-01		AN (F)
*Cupriavidus*	1.67	0.27	6.26	3.76E-10	3.44E-08	0.26	0.27	0.98	3.28E-01	7.42E-01		AE
*Methylobacterium-Methylorubrum*	1.59	0.26	6.16	7.26E-10	4.43E-08	0.48	0.25	1.92	5.51E-02	3.15E-01		AE
*Pseudomonas*	2.41	0.40	6.02	1.77E-09	8.11E-08	0.54	0.39	1.38	1.69E-01	5.49E-01	yes	AE
*Ralstonia*	1.59	0.31	5.21	1.85E-07	6.78E-06	0.46	0.24	1.86	6.23E-02	3.26E-01		AE
*Renibacterium*	2.03	0.39	5.17	2.30E-07	7.00E-06	0.42	0.28	1.53	1.27E-01	5.01E-01		AE
*Stomatobaculum*	-2.92	0.58	-5.03	4.90E-07	1.14E-05	-1.84	0.58	-3.20	1.36E-03	7.58E-02		AN
*Pelomonas*	2.25	0.45	5.03	4.98E-07	1.14E-05	0.20	0.37	0.54	5.92E-01	9.12E-01	yes	AE
*Finegoldia*	2.68	0.57	4.69	2.69E-06	5.47E-05	2.02	0.53	3.81	1.42E-04	1.30E-02	yes	AN
*Streptococcus*	3.47	0.77	4.53	5.94E-06	1.09E-04	2.13	0.70	3.03	2.48E-03	7.58E-02	yes	AN (F)
*Rheinheimera*	1.57	0.35	4.50	6.83E-06	1.14E-04	0.36	0.30	1.22	2.23E-01	6.18E-01		AE
*Diaphorobacter*	1.22	0.28	4.34	1.42E-05	2.17E-04	0.29	0.23	1.25	2.10E-01	5.96E-01		AE
*Caulobacter*	1.37	0.32	4.28	1.90E-05	2.67E-04	0.12	0.34	0.36	7.21E-01	9.20E-01		AE
*Cloacibacterium*	1.87	0.45	4.16	3.15E-05	4.11E-04	0.42	0.34	2.08	3.73E-02	2.84E-01		AN (F)
*Fusobacterium*	-2.78	0.67	-4.15	3.39E-05	4.11E-04	-2.11	0.68	-3.10	1.96E-03	7.58E-02	yes	AN
*Sphingomonas*	1.05	0.25	4.13	3.59E-05	4.11E-04	0.00	0.23	-0.01	9.89E-01	9.89E-01		AE
*Acinetobacter*	2.05	0.50	4.11	3.89E-05	4.19E-04	0.38	0.40	0.96	3.39E-01	7.47E-01		AE
*Cutibacterium*	1.37	0.34	4.01	6.01E-05	6.11E-04	0.46	0.27	1.70	8.94E-02	3.99E-01		AN (AEt)
*Corynebacterium*	2.49	0.66	3.79	1.51E-04	1.39E-03	2.07	0.74	2.81	4.90E-03	8.15E-02	yes	AE
*Solobacterium*	-2.61	0.69	-3.79	1.49E-04	1.39E-03	-2.69	0.69	-3.90	9.59E-05	1.30E-02		AN
*Prevotella*	-1.75	0.51	-3.42	6.33E-04	5.52E-03	-1.50	0.61	-2.45	1.44E-02	1.61E-01	yes	AN
*Lactococcus*	1.65	0.55	2.99	2.79E-03	2.32E-02	0.11	0.42	0.26	7.97E-01	9.34E-01		AN (F)
*Massilia*	1.13	0.38	2.97	3.01E-03	2.39E-02	0.23	0.26	0.86	3.88E-01	8.22E-01		AE
*Peptococcus*	-1.89	0.66	-2.85	4.39E-03	3.35E-02	-2.07	0.72	-2.89	3.81E-03	7.58E-02	yes	AN
*Bulleidia*	-1.14	0.40	-2.80	5.04E-03	3.69E-02	-0.64	0.34	-1.87	6.18E-02	3.26E-01		AN
*Olsenella*	-1.24	0.45	-2.76	5.85E-03	3.83E-02	-0.99	0.38	-2.62	8.92E-03	1.26E-01		AN
*Porphyromonas*	-1.54	0.56	-2.76	5.72E-03	3.83E-02	-0.78	0.63	-1.25	2.12E-01	5.96E-01	yes	AN
*Neisseria*	0.98	0.35	2.78	5.49E-03	3.83E-02	0.27	0.25	1.11	2.65E-01	6.38E-01		AE
*Muribaculum*	0.56	0.21	2.70	6.99E-03	4.30E-02	-0.06	0.23	-0.26	7.93E-01	9.34E-01		AN
*Blautia*	0.86	0.32	2.69	7.04E-03	4.30E-02	0.33	0.41	0.79	4.28E-01	8.49E-01		AN
*Pedobacter*	0.94	0.35	2.66	7.83E-03	4.62E-02	0.03	0.25	0.13	8.95E-01	9.66E-01		AN (F)

Coef-beta coefficient in ANCOM-BC log-linear model, se -standard error, test- statistic – test statistic for beta coefficient, p-value – p-value for beta coefficient, adj p-value – p-value adjusted for multiple comparisons, AN, anaerobic; AE, aerobic; AN (AEt), aerotolerant anaerobic; AN (F), facultatively anaerobic.

**Figure 7:**
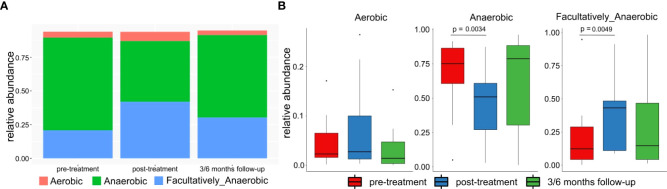
**(A)** Mean relative abundance of anaerobic, aerobic and facultatively anaerobic bacteria in patients pre-treatment, after treatment and in 3/6 months follow-up. **(B)** Differences between abundances of anaerobic, aerobic and facultatively anaerobic bacteria in patients pre-treatment, after treatment and in 3/6 months follow-up.

Significantly higher percentage of anaerobic bacteria was observed in pre-treatment in comparison to post-treatment samples (p-value=0.0034). In contrast, an increased percentage of facultatively anaerobic bacteria was found in post-treatment patient (p-value=0.0049). No significant difference was found for anaerobic and facultatively anaerobic bacteria at follow-up samples. No similar findings were not observed in the aerobic bacteria abundances (**Figures 7A, B**).

## Discussion

Here, we firstly compared the CM of healthy women with those of cervical cancer patients. The CM was dominated by *Lactobacillus* in 12 of 15 premenopausal healthy control samples and in one (a 25-years-old cancer patient in whom the percentage of L*actobacillus* reached only 45.27%) of 5 samples collected from premenopausal cancer patients before treatment, and in 7 of 16 postmenopausal healthy women and 3 of 10 postmenopausal cancer patients. CMs of 10 and 3 of 20 healthy women with the noticeable lactobacilli levels were dominated by *L. crispatus* and *L. iners*, respectively, while all 4 CMs of cancer patients with the noticeable lactobacilli levels were dominated by *L. iners*.

In women with a *Lactobacillus*-dominated CM, vaginal anaerobic metabolism of glycogen to lactic acid acidifies the vagina to a pH of <4.5. The resulting acidic environment defends the vaginal mucosa and cervical epithelium against cervicovaginal dysbiosis and vaginally transmitted infections. A vaginal microbiota dominated by *Lactobacillus* is considered to be a marker of a healthy vagina and vice-versa (8). However, the composition of the vaginal microbiota is affected by various factors, including menstruation, sexual behavior, hygiene habits, lactation, use of antibiotics, prebiotics, and probiotics, and dietary factors (1, 8, 9); even daily dynamic changes in the vaginal microbiome have been reported (10–12). In reproductive-age the predominant factor inhabiting the healthy vagina with lactobacilli is estrogen and, therefore, in postmenopausal women, the decreased levels of estrogen make that lactobacilli are no longer the dominant bacteria of the vaginal microbiome (6, 40, 41). It is also considered that cervicovaginal microbiomes with high levels of *L. crispatus* relate to healthy individuals, while those dominated by *L. iners* may relate to cervical cancer alone (6). Our findings indicate that a shift to a polymicrobial CM in both pre- and postmenopausal patients likely relates to cervical cancer.

While cervical mucosal community type (CT) dominated by several sub-genera, and *Lactobacillus* OTUs was associated with CIN 2+ (42), lactobacilli did not differentiate patients with CIN2:3-CC from controls (43); instead, an unclassified species within the genus Gardnerella was more abundant in the control group (43). In BV, the most abundant bacteria belong to the genera *Corynebacterium*, *Atopobium*, *Gardnerella*, *Mobiluncus*, *Peptoniphilus*, *Anaerococcus, Prevotella*, *Bacteroides*, *Finegoldia*, *Sneathia*, *Lachnospiraceae taxon Lachnovaginosum genomospecies* (BVAB1), *Eggerthella*, and several under-represented members of *Streptococcus*, *Atopobium*, *Megasphaera*, *Leptotrichia*, and *Staphylococcus* (1, 38, 44–47). Decreased abundance of *Lactobacillus* compromises the acidic vaginal environment, leading to impairment of cervicovaginal mucosal defense mechanisms. Thus, healthy cervicovaginal microbiomes promote health, while disrupted local immune regulation and inflammation in BV are associated with cervicovaginal pathologies such as HPV infection and gynecological cancers (4, 13–19).

A study examining the association between the CM and high-grade cervical intraepithelial neoplasia (CIN 2+) in women infected with high-risk HPV revealed that the alpha and beta diversity of the vaginal microbiota were not associated with either CIN severity or oxidative DNA damage (42). In addition, a case-control analysis (43) of the CM in healthy subjects and patients diagnosed with cervical intraepithelial neoplasia grade 2/3 or invasive cancer (CIN2/3-CC) showed that microbial richness was significantly higher in the CIN2/3-CC group than in the control group, accompanied by an increase in the number of operational taxonomic units (OTUs). Conversely, the diversity index, calculated using the Shannon, Simpson, and Bray–Curtis indices, did not differ significantly between the groups at the phylum or genera levels. Other studies show that bacterial diversity did not differ between healthy individuals and patients with CIN2/3-CC cancer (18, 42). By contrast, species richness was higher in the pre-cancerous and CIN2/3-CC groups than in the control group. Thus, the microbial richness can be considered as an indicator of vaginal health, and variations in diversity may be associated with atypical health conditions (48). In this study, we showed that the microbiome diversity was significantly higher in pre-treatment than in control samples (the Shannon index), but there were no significant differences in species richness (the Chao index). Due to the relatively small groups of our pre- and postmenopausal cancer patients, we did not undertake further analyzes taking into account the FIGO staging, since it resulted in excessive patient stratification. Moreover, probably for similar reasons, no analyzes of the cervical microbiome in relation to the cervical cancer stage were performed in any of the previously published studies.

Therefore, our findings are only partly consistent with those reported in other studies (18, 42, 43, 48). The main differences relate to diversity and richness. While we found that the CM diversity in pre-treatment cancer samples was significantly higher than that in control samples and that there were no significant differences regarding species richness, earlier studies report the opposite. In addition, we detected lactobacilli depletion and accompanied increased abundance of anaerobic bacteria in women with cervical cancer only in premenopausal patients, while lactobacill depletion and anaerobes abundance did not differ between postmenopausal patients and controls. These findings are in accordance with prior studies (49, 50), where increased abundance of anaerobic bacteria were detected in women with cervical disease.

CRT and RT are curative or palliative therapies for gynecologic cancer that may disturb the composition of the vaginal microbiome (21). We showed that 31 genera, of which *Streptococcus, Prevotella, Fusobacterium, Porphyromonas*, and *Finegoldia* were the most abundant, differentiated pre- and post-radiation samples, and only 2 differentiated pre-radiation and follow-up samples. However, neither microbiome diversity nor microbiome richness differentiated pre-treatment samples from post-treatment samples, either at the end of the therapy or 3 and 6 months later.

A previous pilot study (4) showed a strong reduction in cervical bacterial load after RT. Just like in our study, neither alpha nor beta diversity differed significantly when pre-radiation with post-radiation samples were compared, which, as suggested, might be due to the small number of samples and inter-individual differences in CM profiles. Another study (22) comparing the vaginal microbiome in gynecological cancer patients before and at 1–2 months after RT reported that most post-RT microbiota communities did not overlap with normal microbiomes. In addition, there was a trend toward lower microbial richness in healthy samples relative to gynecological cancer samples, and 13 phylogroups on the genus level differentiated pre-RT samples from post-RT samples. A recently published study (51) reported significant differences in the vaginal microbiome between patients with cervical cancer receiving pelvic CRT and healthy controls, and again, no significant differences in bacterial richness and alpha and beta diversity between radiation treatment time points. However, the relative abundances of *Gammaproteobacteria*, *Gemmatimonadetes*, *Gemmatimonadales*, *Pseudomonadales*, *Gemmatimonadaceae*, *Rikenellaceae*, *Acinetobacter*, *Desulfovibrio*, *Prevotella 9*, *Rikenellaceae RC9 gut group*, and *Turicibacter* increased with time.

Although there are some inconsistencies between our results and those mentioned above (4, 21), the presence of cervical cancer-related dysbiosis is obvious and the association between BV and cancer risk is well documented (18, 39). Nevertheless, our understanding of the role of cervicovaginal dysbiosis in the development and progression of gynecological cancer is limited. Therefore, we can only speculate that chemoradiation and radiation treatment trigger a synergistic effect between cervicovaginal dysbiosis and immune activation (4). However, it cannot be ruled out that the changes in the cervical microbiome partly resulted from the presence of tumor necrosis. Most recipients of pelvic radiotherapy develop alterations in the overall composition of the gastrointestinal microbiome, which are associated with decreased gut microbiome diversity (52, 53). In turn, although changes in the CM after chemoradiation treatment for cervical cancer might offer insight into new therapeutic options in the field of gynecological oncology (4), further studies are needed to evaluate this. Research should include larger and diverse groups of women with gynecologic cancers and similarly various groups of healthy women.

## Conclusions

While previously published studies evaluated mostly the impact of chemoradiation treatment on the cervicovaginal microbiome (1, 4, 51, 54), our study for the first time compared also cervical microbiota of cancer patients and healthy women divided into pre- and postmenopausal groups. Other studies either did not include comparisons with healthy controls or the control groups were sparse and analyses did not take into account the hormonal status of the women from the compared groups. Despite limitations, which include the small size of the studied groups, reduced number of the follow-up samples taken from cancer patients and lack of information whether and how the HPV status could affect the cervical microbiome in our cancer patients, the study allowed for several conclusions. While the cervicovaginal microbiome is required to maintain vaginal homeostasis, analysis of the bacterial 16S rRNA gene revealed significant changes in the CM of cervical cancer patients shifting toward polymicrobial overgrowth, which is accompanied by a reduced abundance of *Lactobacillus*, at least in premenopausal patients. Our data provide additional evidences on differences in microbiome composition between pre- and postmenopausal healthy women as well as between pre- and post-treatment cancer samples, which may relate to CRT.

However, further research is needed to determine whether alteration of the cervicovaginal microbiome may offer new therapeutic options leading to a promising strategy. If so, identification of cervicovaginal dysbiosis, related to gynaecological cancers, can be considered an interesting field of investigation.

## Data availability statement

The datasets presented in this study can be found in online repositories. The names of the repository/repositories and accession number(s) can be found below: https://www.ebi.ac.uk/ena, PRJEB43410.

## Ethics statement

The studies involving human participants were reviewed and approved by Bioethics Committee at the Maria Sklodowska-Curie National Research Institute of Oncology (69/2017 dated 06.07.2017). The patients/participants provided their written informed consent to participate in this study.

## Author contributions

Conceptualization, JO and MB; methodology, JO; software, MK; formal analysis, MK and JO, investigation, NZ-L and AP; resources, MB, BL, RK, and AN; data curation, MK; writing—original draft preparation, JO; writing—review and editing, MK and NZ-L; visualization, MK; supervision, JO; project administration, NZ-L; funding acquisition, JO. All authors have read and agreed to the published version of the manuscript.

## Funding

This work was financed by the National Science Center [2017/27/B/NZ5/01504].

## Conflict of interest

The authors declare that the research was conducted in the absence of any commercial or financial relationships that could be construed as a potential conflict of interest.

## Publisher’s note

All claims expressed in this article are solely those of the authors and do not necessarily represent those of their affiliated organizations, or those of the publisher, the editors and the reviewers. Any product that may be evaluated in this article, or claim that may be made by its manufacturer, is not guaranteed or endorsed by the publisher.

## Supplementary material

Supplementary Table 1 Bacterial counts from MEGAN used to generate.

Supplementary Table 2 Results of comparison between healthy controls and post-treatment patients.
